# Carotid body chemoreceptors, sympathetic neural activation, and cardiometabolic disease

**DOI:** 10.1186/s40659-016-0073-8

**Published:** 2016-02-26

**Authors:** Rodrigo Iturriaga, Rodrigo Del Rio, Juan Idiaquez, Virend K. Somers

**Affiliations:** Laboratorio de Neurobiología, Facultad de Ciencias Biológicas, Pontificia Universidad Católica de Chile, Santiago, Chile; Laboratory of Cardiorespiratory Control, Centro de Investigación Biomédica, Universidad Autónoma de Chile, Santiago, Chile; Dirección de Investigación, Universidad Científica del Sur, Lima, Peru; Catedra de Neurología, Escuela de Medicina, Universidad de Valparaíso, Valparaíso, Chile; Division of Cardiovascular Diseases, Mayo Clinic, Rochester, MN USA

**Keywords:** Autonomic dysfunction, Carotid body, Heart failure, Metabolic syndrome, Obstructive sleep apnea, Sympathetic activation

## Abstract

The carotid body (CB) is the main peripheral chemoreceptor that senses the arterial PO_2_, PCO_2_ and pH. In response to hypoxemia, hypercapnia and acidosis, carotid chemosensory discharge elicits reflex respiratory, autonomic and cardiovascular adjustments. The classical construct considers the CB as the main peripheral oxygen sensor, triggering reflex physiological responses to acute hypoxemia and facilitating the ventilatory acclimation to chronic hypoxemia at high altitude. However, a growing body of experimental evidence supports the novel concept that an abnormally enhanced CB chemosensory input to the brainstem contributes to overactivation of the sympathetic nervous system, and consequent pathology. Indeed, the CB has been implicated in several diseases associated with increases in central sympathetic outflow. These include hypertension, heart failure, sleep apnea, chronic obstructive pulmonary disease and metabolic syndrome. Indeed, ablation of the CB has been proposed for the treatment of severe and resistant hypertension in humans. In this review, we will analyze and discuss new evidence supporting an important role for the CB chemoreceptor in the progression of autonomic and cardiorespiratory alterations induced by heart failure, obstructive sleep apnea, chronic obstructive pulmonary disease and metabolic syndrome.

## **Background**

The carotid body (CB) is a polymodal chemoreceptor located in the carotid bifurcation, which is activated by hypoxemia, hypercapnia, acidosis, reduction of arterial blood flow, temperature change and low levels of glucose [1–4]. Reflex cardiorespiratory responses are characterized by hyperventilation and increased sympathetic discharge to the vascular beds and the heart. Tachycardia associated with hyperventilation in turn augments cardiac output, acutely raising arterial blood pressure. The CB chemoreceptor (glomus or type I) cells, which make synaptic contact with the nerve terminals of the chemosensory petrosal neurons, are considered the sensors of the natural stimuli [1–4]. The current model of CB chemoreception holds that hypoxia and hypercapnia-acidosis close voltage independent (TASK) and voltage-dependent K^+^ channels, leading to glomus cell depolarization, entry of Ca^2+^ through L-type Ca^2+^ channels, and release of one or more excitatory transmitters, which increases the discharges of the nerve endings of chemosensory neurons [2–5]. Several molecules are present in glomus cells, but acetylcholine and adenosine triphosphate fulfill most of the criteria to be considered as excitatory transmitters between the glomus cells and petrosal nerve endings [3–5]. However, other molecules such as dopamine, histamine, nitric oxide (NO), carbon monoxide, H_2_S, and endothelin-1 (ET-1) modulate the chemosensory process by producing tonic actions on CB blood vessels or direct effects on glomus cells [3–5]. More recently, pro-inflammatory cytokines such as interleukin 1β, interleukin 6 and TNF-α have been found to modulate CB chemoreception in rats [6–8].

The classical physiological paradigm considers the CB as the main oxygen sensor, triggering ventilatory responses to acute hypoxemia and modulating the ventilatory acclimation to high altitude. Notwithstanding, a growing body of evidence involved the CB in several sympathetic-mediated human diseases [8–17]. Indeed, selective ablation of the CB improves survival in heart failure (HF) experimental models [10, 11], prevents the development of insulin resistance and hypertension in rats fed with a high fat diet [18], and attenuates the hypertension induced by chronic intermittent hypoxia in a rat model of obstructive sleep apnea [19].

## The carotid body and heart failure

Heart failure (HF) is characterized by frequent hospitalizations and high mortality risk. Accordingly, HF is considered a major health problem, affecting 20 % of the adult population [20, 21]. The pathophysiology of HF is characterized by a progressive decrease in cardiac function, which severely impacts the blood supply to several vascular beds [22–24]. Two main characteristics of HF are the presence of autonomic imbalance and disordered breathing patterns, both of which has been shown to be strongly associated with the degree of cardiac failure [11, 25–27]. Indeed, the contribution of heightened CB chemoreflex drive and excessive sympathetic outflow to the development and progression of HF has been demonstrated in both humans and experimental animal models [13, 27, 28]. Indeed, an enhanced CB chemoreflex drive has been shown to play a key role in the progression of cardiorespiratory disorders in HF [11, 29], and high CB chemosensitivity correlates strongly with high mortality risk and poor prognosis in patients with HF [30]. In experimental HF, the CB chemosensory activity is tonically elevated leading to sympatho-excitation and destabilization of breathing [13, 27, 31].

**Fig. 1:**
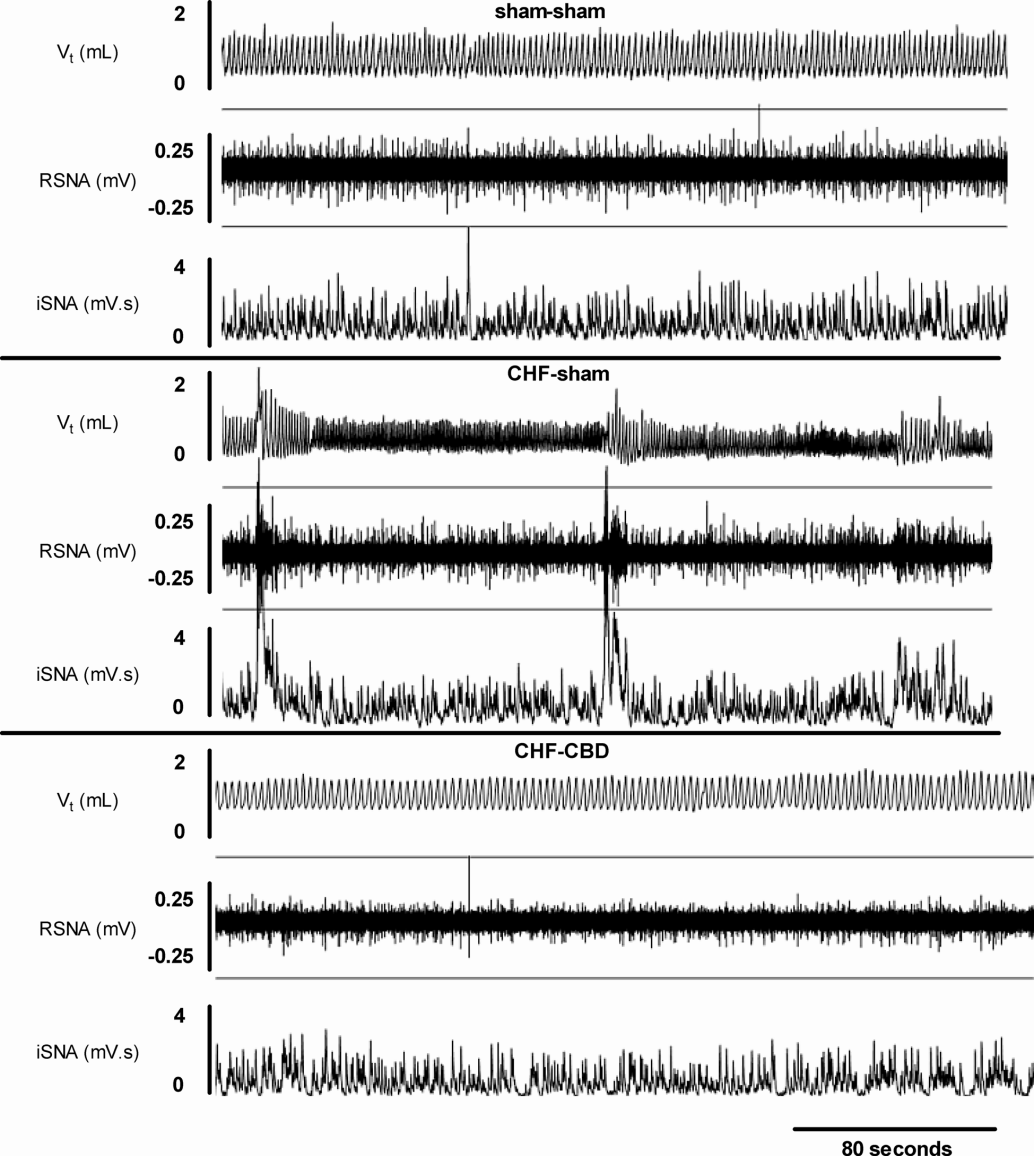
Carotid body mediates sympathoexcitation and oscillatory breathing patterns in heart failure. Representative recordings of tidal ventilation (Vt), renal sympathetic nerve activity (RSNA) and integrated RSNA (iSNA) in one control (sham–sham) animal, one chronic heart failure animal with intact carotid bodies (CHF–sham) and one chronic heart failure animal that underwent carotid body denervation (CHF–CBD). Note that CBD normalize RSNA and ventilatory oscillations. Reprinted from Marcus et al. [27] with permission of John Wiley and Sons

**Fig. 2:**
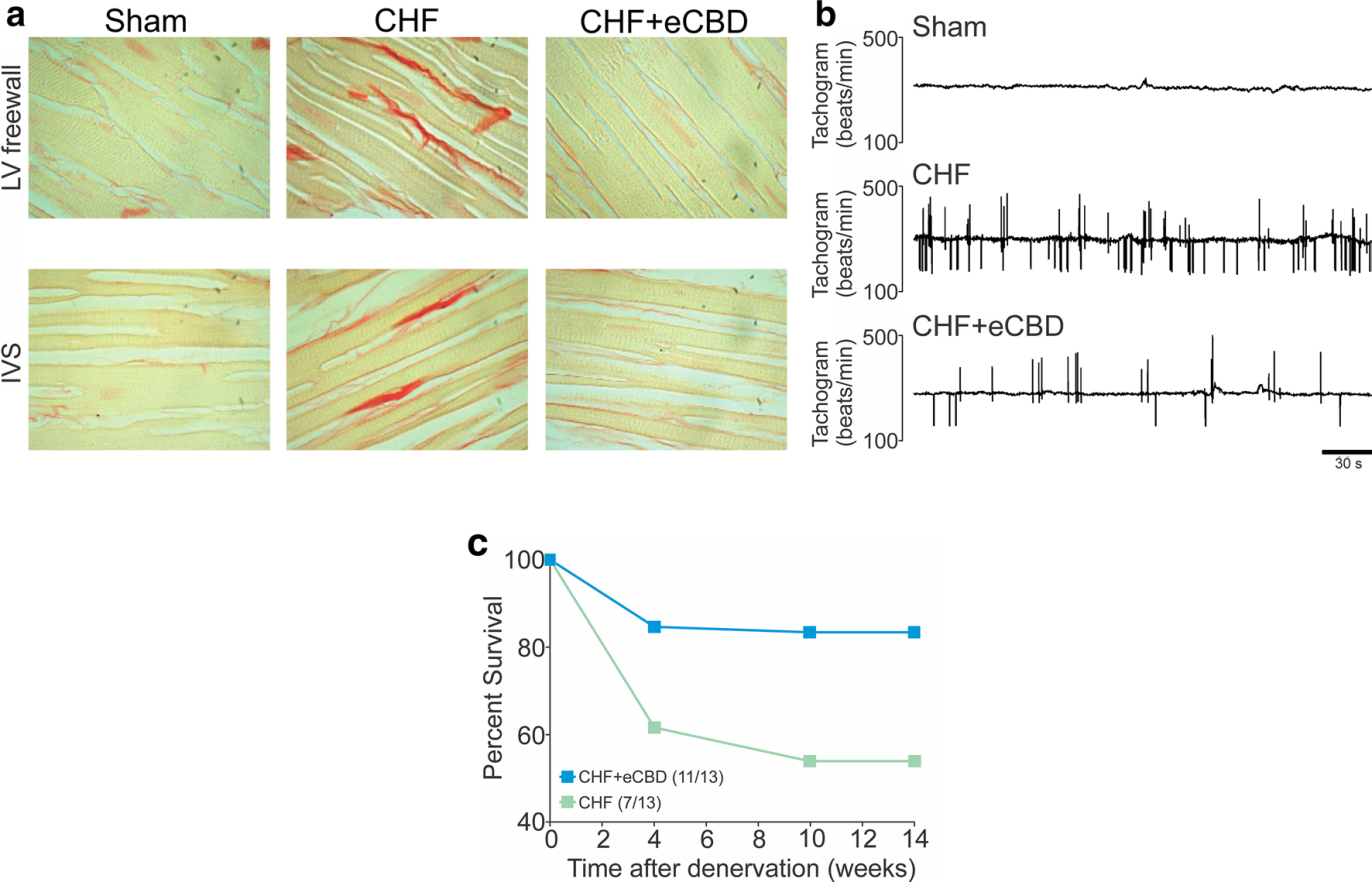
Carotid Body denervation reduced myocardial fibrosis and cardiac arrhythmias and improved survival in heart failure rats. **a** Rats with chronic heart failure (CHF) and with CHF and carotid body denervation (eCBD) displayed tissue fibrosis in noninfarcted areas. The left ventricle (LV) freewall and the interventricular septum (IVS) obtained from CHF rats showed a marked collagen deposition. Selective eCBD significantly reduced cardiac fibrosis in the LV-freewall and in the IVS. **b** Representative tachograms showing arrhythmic episodes in a CHF rat and a marked decreased in the arrhythmic events in CHF+eCBD rat. **c** Rats that underwent eCBD showed reduced mortality rate compared to CHF rats with functional CBs. Modified from Del Rio et al. [10], with permission of Elsevier

The physiological mechanisms underlying cardiorespiratory alterations in HF are not fully understood. The most widely accepted model of CB chemoreflex regulation states that chemosensory nerve fibers from the CB project to the nucleus tractus solitarius (NTS), which integrates the CB afferent visceral sensory input. The NTS in turn sends efferent projections to the respiratory neuronal network and brainstem autonomic sympathetic nuclei, such as the rostral ventrolateral medulla (RVLM) [32]. It has been shown that in HF rats, the CB chemosensory discharge in normoxia is enhanced resulting in hyper-activation of pre-sympathetic neurons located in the RVLM, which finally leads to increased central sympathetic outflow [33]. Furthermore, Marcus et al. [27] showed that an augmented CB afferent activity triggered respiratory-sympathetic coupling in rabbits with HF. Notably, selective ablation of the CB chemoreceptors decreased RVLM pre-sympathetic neuron activation, restored normal sympathetic outflow, and markedly reduced the incidence of oscillatory breathing patterns in HF (see Fig. 1). Additionally, if performed early during the progression of HF, CB ablation reduced collagen deposition and fibrosis in the ventricular myocardium, decreased the number of cardiac arrhythmias, blunted cardiac function deterioration and strikingly improved survival of HF rats (Fig. 2). Together, these results strongly support a crucial role of the CB in the development of abnormal breathing patterns and increased sympathetic outflow, adding more stress to the failing heart, and ultimately leading to higher mortality risk.

The tonic hyper-activation of the CB chemoreceptor cells during HF appears to be a key step in disease progression. Identifying molecular mechanisms underlying CB chemoreceptor activation could therefore lead to novel future interventions intended to normalize CB chemosensory activity in HF. Recently, Schultz and colleagues provided a comprehensive review on the plausible mechanisms underpinning the enhanced CB function in HF [34]. Due to the nature of HF, a chronic reduction in blood supply to several organs is expected during the progression of the disease. Thus, it has been hypothesized that blood flow restrictions to the CB region could play a role in inducing augmented CB chemosensory discharge in HF. Ding et al. [35] provide the first evidence showing that chronic blood flow restrictions to the CB in normal animals induced CB chemorelfex activation. Indeed, reducing blood supply to the CB to a similar extent to flows observed in HF animals results in CB chemosensory potentiation and increases in renal sympathetic nerve activity [35]. Recently, the blood flow-sensitive transcription factor, Kruppel-like factor 2 (KLF-2), was shown to be constitutively expressed in the CB, particularly in chemoreceptor cells [36]. Furthermore, KLF-2 expression in the CBs is markedly reduced during HF [36]. The viral transduction of KLF-2 in the CB of rabbits with HF normalizes CB function and reduces sympathetic outflow despite the chronic reduction of blood flow to the tissue [34]. In addition, other factors such as decreases in NO bioavailability and increases in local and systemic levels of angiotensin II also could contribute to an increased CB chemoreflex drive in HF. Furthermore, oxidative stress has been related to augmented CB discharge in HF, since genetic manipulation to increase superoxide dismutase within the CB tissue significantly reduced CB afferent activity [35]. Further studies are needed to uncover the role played by KLF-2 in the regulation of NO synthase expression as well as superoxide dismutase expression and local angiotensin II production in the CB during HF.

## The carotid body and obstructive sleep apnea

Obstructive sleep apnea (OSA) is elicited by repeated total or partial occlusions of the upper airway, and is associated with daytime sleepiness, fatigue, depressed mood, and cognitive alterations [37, 38]. Several epidemiological studies have demonstrated that OSA is an independent risk factor for the development and progression of systemic hypertension, showing a positive correlation between the apnea/hypopnea index and the severity of hypertension [38–41]. The repeated episodes of airflow obstruction during sleep produces cyclic intermittent hypoxemia and hypercapnia, which stimulate the CB eliciting reflex ventilatory, sympathetic and hypertensive responses. Among these disturbances, chronic intermittent hypoxemia (CIH) is considered the main factor related to systemic hypertension [17, 42–48].

Although the link between OSA and hypertension is well established, the pathogenic mechanisms underlying the onset and maintenance of the hypertension are not entirely known. It has been proposed that CIH elicits oxidative stress, inflammation, and sympathetic hyperactivity, which leads to endothelial dysfunction and hypertension [8, 17, 43, 49, 50]. However, studies performed in OSA patients are limited by degree of invasiveness, and because OSA patients often present concomitant co-morbidities (such as obesity and metabolic alterations), which themselves increase cardiovascular risk. Therefore, casual relationships between OSA and associated disease conditions are not well demarcated. On the other hand, experimental models of rodents exposed to CIH reproduce several cardiovascular pathologic features of OSA including hypertension and sympathetic hyperactivity [8, 19, 51–54, 56–62].

**Fig. 3:**
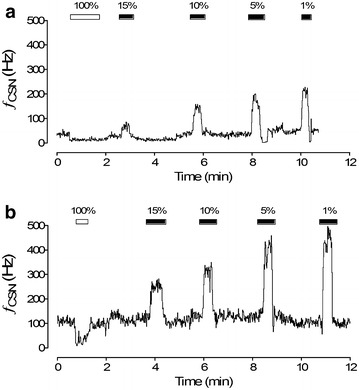
CIH increased basal carotid chemosensory discharges and induced a potentiation of chemosensory responses to acute hypoxia. The chemosensory responses to various levels of inspired O_2_ (PO_2_ ≈ 100−1 %) were measured from one carotid sinus nerve of a sham rat (**a**) and from a rat exposed to cyclic hypoxic episodes (PO_2_ to 35 mmHg, 12 times per h during 8 h) for 21 days. (**b**) ƒ_csn_, frequency of carotid chemosensory discharges expressed in Hz. Rats were anesthetized with sodium pentobarbitone (40 mg/kg ip) and breathed spontaneously room air. Reprinted from Iturriaga et al. [8] with permission of John Wiley and Sons

Patients with recently diagnosed OSA show enhanced vasopressor and ventilatory responses to acute hypoxemia [63–66], and manifest sympathetic hyperactivity evidenced by an increased muscle sympathetic neural activity [67] and higher levels of urinary norepinephrine [68]. Similarly, rodents exposed to CIH have enhanced cardiorespiratory and sympathetic responses to hypoxia, and develop systemic hypertension [5, 69–74]. Furthermore, both OSA patients and animals exposed to CIH show reduction of baroreflex gain and alterations of heart rate variability towards a predominance of sympathetic drive [51, 65, 75–79]. Thus, it is likely that enhanced sympathetic activity along with reduction of baroreflex gain may contribute to the rise of arterial blood pressure following CIH. The enhanced cardiorespiratory responses to acute hypoxemia found in patients with recently diagnosed OSA has been attributed to an enhanced hypoxic chemoreflex [63–66, 80], suggesting that the CB is involved in the pathological alterations induced by OSA. Although Fletcher et al. [19] found that CB denervation prevented hypertension in rats exposed to CIH, the contribution of the CB to the cardiovascular pathology induced by OSA was not considered. However, in the last decade the proposal that an abnormally enhanced CB chemosensory drive is involved in the progression of CIH-induced hypertension has received substantial attention [43, 49, 58, 81]. Recordings of CB neural discharges in situ and in vitro have demonstrate that CIH selectively increases basal discharge in normoxia, and potentiates the chemosensory responses to acute hypoxia [18, 51, 52, 54–59, 78, 79] (see Fig. 3).

Reactive oxygen species (ROS) and reactive nitrogen species (RNS) have been implicated as mediators of cardiovascular and cognitive alterations in OSA patients [44, 82–85] and animal models [51, 52, 54, 55, 57, 59]. We tested the hypothesis that oxidative stress contributes to CB chemosensory potentiation and the progression of the hypertension in rats exposed to CIH [51]. We found that CIH increased plasma lipid peroxidation and formation of the oxidative stress marker 3-nitrotyrosine (3-NT) in the CB. In addition, CIH enhanced CB chemosensory and ventilatory responses to hypoxia and elicited hypertension. Antioxidant treatment with ascorbic acid reduced the increased systemic and CB oxidative stress, normalized CB chemoreflex function, and markedly reduced the elevated arterial blood pressure. Although, these results strongly suggest that CB chemosensory potentiation is mediated by oxidative stress, it is a matter of debate as to whether ROS *per se* may increase CB chemosensory discharge [86]. Thus, it is likely that other molecules downstream of ROS signaling mediate the CIH-induced effects of ROS on CB chemoreception. Among the molecules upregulated in the CB by CIH, such as ET-1, VEGF and iNOS [52, 53, 79, 87, 89], pro-inflammatory cytokines have been proposed as mediators of the CB chemosensory potentiation induced by CIH [8, 9, 52, 54, 87, 88, 89]. We found that CIH induced a ROS-dependent increase in TNF-α and IL-1β levels in the CB, suggesting that these pro-inflammatory cytokines may mediate the ROS-induced CB potentiation [51, 52]. Furthermore, ibuprofen treatment prevented CB cytokine overexpression, as well as the enhanced hypoxic ventilatory response and the hypertension, but failed to block the enhanced CB chemosensory responses [54]. Thus, our studies suggest that upregulation of TNF-α and IL-1β in the CB induced by CIH is linked to oxidative stress, as well as to the enhanced CB chemosensory responsiveness to hypoxia, but the chemosensory potentiation does not depend on the increased TNF-α and IL-1β levels in the CB. However, pro-inflammatory cytokines contribute to enhance the hypoxic ventilatory response and to the hypertension induced by CIH, suggesting that multiple mechanisms may participate in the cardiorespiratory alterations induced by CIH.

## The carotid body and chronic obstructive pulmonary disease

Chronic obstructive pulmonary disease (COPD) is a systemic disease that includes many extra pulmonary manifestations including systemic inflammation, cachexia, and muscle dysfunction [90]. Increased sympathetic activation in COPD is evident from increased plasma norepinephrine, and associated with increased plasma renin activity and aldosterone concentration [90]. Heightened muscle sympathetic nerve activity occurs in patients with chronic respiratory failure [91–93]. In COPD patients, coexistent hypoxemia and hypercapnia, activates peripheral chemoreceptors (hypoxemia) and central chemoreceptors (hypercapnia). Short-term oxygen supplementation reduces muscle sympathetic nerve traffic in these patients [91] suggesting that peripheral chemoreceptors are involved. Sympathetic activation in COPD may be also related to other conditions like arterial and cardiac baroreflex dysfunction, breathing patterns and metaboreflex excitation [94]. Indeed, slow breathing causes a drop of sympathetic overactivity in COPD [95], possibly improving baroreflex sensitivity and gas exchange. The peripheral chemoreceptors therefore are likely contributors to elevated muscle sympathetic nerve discharge in COPD [92].

## Metabolic alterations: a new role for the carotid body?

Metabolic syndrome is a growing health problem worldwide, with a high prevalence and strong associations with cardiovascular risk and diabetes. Autonomic dysfunction, characterized by sympathetic hyperactivity, vagal impairment, and impaired baroreflex sensitivity are characteristics of the metabolic syndrome and of disease conditions where the CB may be implicated, such as hypertension [96–99]. In addition, patients with metabolic disorders also have increased levels of leptin, ROS and pro-inflammatory cytokines. It is conceivable that CB chemosensory function may be compromised in the metabolic syndrome. In fact, it is known that obesity increases adipokine levels (i.e. leptin, resistin, TNF-α and IL-6), which in turn may activate NADPH oxidase increasing superoxide radical production. Superoxide reacts with NO to form peroxynitrite, decreasing NO availability, which contributes to the endothelial dysfunction [100]. We found a marked increase of 3-NT in the CB from rats exposed to CIH, which correlates with the enhanced chemosensory responses to hypoxia [51, 52], supporting the idea that oxidative-nitrosative stress plays a critical role in CB chemosensory potentiation induced by CIH [8, 9, 52, 53].

The notion that the CB is involved in the metabolic regulation of glucose and insulin is not new [12, 101]. Recently, Ribeiro et al. [18] studied the role played by the CB in a rat model of insulin resistance induced by high fat-sucrose diets. They found that CB stimulation by insulin seems to be involved in the development of insulin resistance and hypertension. Bilateral CB denervation prevents diet-induced insulin resistance and hypertension, suggesting that insulin-induced CB chemosensory excitation is responsible for the increased sympathetic outflow, creating a positive feedback, which results in severe insulin resistance and hypertension. Furthermore, Porzionato et al. [14] suggested that the CB chemoreceptors are a link between the metabolic disorders and the effects of CIH. Indeed, they proposed that “hyperleptinemia and CIH may be interrelated mechanisms of sympathoactivation through peripheral chemoreceptors, because CIH increases plasma leptin levels and leptin immunoreactivity in the CB”. Recently, Trombetta el al. [102] found evidence that metabolic disorders may interact with OSA to potentiate the hypoxic chemoreflexes in humans. In fact, they reported that OSA patients with metabolic syndrome had a higher ventilatory response to hypoxia, and higher muscle sympathetic basal discharge in normoxia and in response to hypoxia, than OSA patients without a metabolic disorder, suggesting that metabolic syndrome could enhanced the CB chemoreflex drive. Similarly, Fenik et al. [103] found that rats exposed to CIH for 35 days showed progressively reduced responses to insulin, which disappeared after 35 days of normoxic exposure. They also found that treatment with losartan eliminated the effects of CIH on insulin release, suggesting a role for the angiotensin-catecholaminergic pathway. Although these new and provocative ideas suggest that metabolic alterations may enhance CB chemosensory responses to hypoxia and increase sympathetic outflow, direct recordings of CB chemosensory discharge in metabolically altered models are required to determine if metabolic alterations in and of themselves, do indeed increase CB chemosensory activity.

## Conclusions

In summary, the available evidence suggests that the CB contributes to the development of autonomic alterations. Identification of those pathways underlying the contribution of the CB to hypertension induced by CIH will provide new insights into the pathogenesis of the cardiovascular alterations observed in OSA and other disease conditions. Furthermore, a mechanistic understanding of altered CB function in sympathetic-mediated diseases will be relevant to improve current treatment options and to develop new therapeutic strategies intended to reduce human disease progression.

## Abbreviations

CB: carotid body
CODP: chronic obstructive pulmonary disease
ET-1: endothelein-1
HF: heart failure
iNOS: inducible nitric oxide synthase
IL-6: interleukin 6
IL-1β: interleukin 1 β
KLF-2: Krüppel-like factor 2
NO: nitric oxide
NTS: nucleus tractus solitarius
3-NT: 3-nitrotyrosine
OSA: obstructive sleep apnea
PO_2_: oxygen partial pressure
PCO_2_: carbon dioxide partial pressure
ROS: reactive oxygen species
RNS: reactive nitrogen specie
RVLM: rostral ventrolateral medulla
TNF-α: tumor necrosis factor-α
VEGF: vascular endothelial growth factor
